# Exosomes as Emerging Pro-Tumorigenic Mediators of the Senescence-Associated Secretory Phenotype

**DOI:** 10.3390/ijms20102547

**Published:** 2019-05-24

**Authors:** Rekha Jakhar, Karen Crasta

**Affiliations:** 1 Lee Kong Chian School of Medicine, Nanyang Technological University Singapore, 59 Nanyang Drive, Singapore 636921, Singapore; rekhajakhar@ntu.edu.sg; 2 School of Biological Sciences, Nanyang Technological University Singapore, 59 Nanyang Drive, Singapore 636921, Singapore; 3 Agency of Science, Technology and Research, Institute of Molecular and Cell Biology, Singapore 636921, Singapore

**Keywords:** senescence, SASP, pro-tumourigenic, exosomes, cancer

## Abstract

Communication between cells is quintessential for biological function and cellular homeostasis. Membrane-bound extracellular vesicles known as exosomes play pivotal roles in mediating intercellular communication in tumor microenvironments. These vesicles and exosomes carry and transfer biomolecules such as proteins, lipids and nucleic acids. Here we focus on exosomes secreted from senescent cells. Cellular senescence can alter the microenvironment and influence neighbouring cells via the senescence-associated secretory phenotype (SASP), which consists of factors such as cytokines, chemokines, matrix proteases and growth factors. This review focuses on exosomes as emerging SASP components that can confer pro-tumorigenic effects in pre-malignant recipient cells. This is in addition to their role in carrying SASP factors. Transfer of such exosomal components may potentially lead to cell proliferation, inflammation and chromosomal instability, and consequently cancer initiation. Senescent cells are known to gather in various tissues with age; eliminating senescent cells or blocking the detrimental effects of the SASP has been shown to alleviate multiple age-related phenotypes. Hence, we speculate that a better understanding of the role of exosomes released from senescent cells in the context of cancer biology may have implications for elucidating mechanisms by which aging promotes cancer and other age-related diseases, and how therapeutic resistance is exacerbated with age.

## 1. Introduction

Cellular senescence is a cellular stress response that culminates in a state of stable cell cycle arrest [1]. As such, it has long been thought to function as an anti-proliferative mechanism against tumor formation in cancers. Senescence has also been strongly associated with age-associated diseases, and has been implicated in developmental processes and wound healing [2]. Cells undergo cellular senescence in response to stressful conditions such as DNA damage, oxidative stress, telomere attrition, oncogenic stress, irradiation and hypoxia. Importantly, the secretion of exosomes has been shown to increase under these conditions [3,4]. This is exciting as exosomes contain proteins, lipids, microRNAs, mRNA and DNA, and can act as “messengers” from one cell to another. This also implies a role for exosomes as senescence effectors. Under stressful conditions, exosomes could relay intercellular cell non-autonomous communication to neighbouring cells and thereby determine the appropriate cell fate response.

The main focus of this review aims to discuss the emerging cell non-autonomous role of senescence-derived exosomes and its possible implications for tumorigenesis. We first take a look at exosomal biogenesis and their functional roles upon uptake in premalignant and cancer cells. We then highlight the role of exosomes during senescence, with a key focus on exosomes as constituents of the senescent secretome known as senescence-associated secretory phenotype (SASP). Lastly, we provide an overall perspective as well as speculate on the implications of exosomes as pro-tumourigenic SASP in the “aging-cancer” nexus.

## 2. Exosome Biogenesis, Composition and Uptake

**Figure 1:**
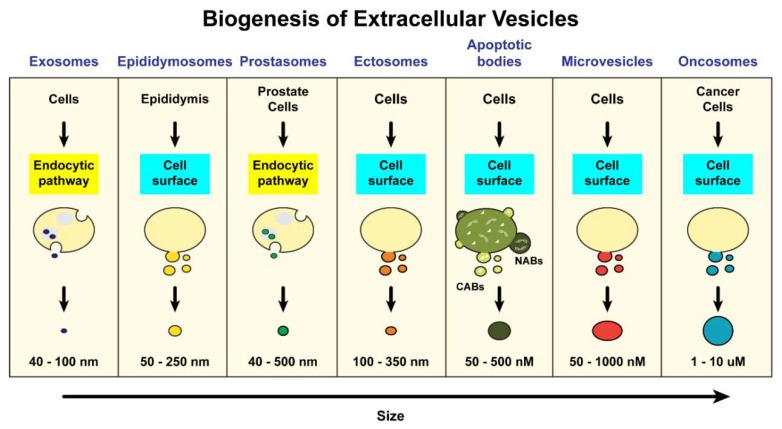
Origin and biogenesis of different groups of extracellular vesicles. EVs are arranged by increasing size from left to right. Left- Exosomes, are secreted by a variety of cell types and are formed in MVBs via the endocytic pathway. Epididymosomes and Prostasomes are EVs found in seminal fluid. Epididymosomes are secreted by cells in the epididymis through budding from the plasma membrane and prostasomes are secreted by epithelial cells of the prostate gland via endosome formation and release into the prostatic fluid. Ectosomes, like exosomes, are secreted by a variety of cell types but unlike exosomes, they are formed via budding from the plasma membrane. Apoptotic bodies are the results of blebs arising from disassembly of apoptotic cells. They are subdivided into two groups, depending on their contents: nuclear (DNA carrying) apoptotic bodes (NABs) and cytoplasmic apoptotic bodies (CABs). Microvesicles are larger in size and are also secreted by a variety of cells. They are also generated by outward budding from the plasma membrane. Oncosomes are much larger than most extracellular vesicles are secreted by various cancer cells via membrane shedding.

Extracellular vesicles are membrane-bound vesicles released by multiple cell types that include immune cells, prostate epithelial cells, stem cells, cancer cells, and neurons [5]. These include exosomes, epididymosomes, prostasomes, ectosomes, apoptotic bodies, microvesicles, and more recently oncosomes (Figure 1). Even though perplexity exists between the term “exosome” and “microvesicles”, these can be distinguished on the basis of their sizes, functional properties and biogenesis (Figure 1).

**Figure 2:**
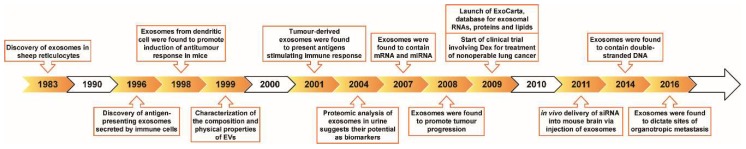
Historic landmarks for the discovery and application of exosomes. Chronological summary of the key events that led to the discovery and application of exosomes and EVs from 1983 to 2016.

Exosomes are basically nano-sized (ranging from 40–100 nm) intercellular communication shuttles. Since the discovery of exosomes in 1983, it has become evident that exosomes contribute to many aspects of physiology and disease, mainly via cell-to-cell communication [5]. We highlight a few exciting milestones in the biology of exosome research in Figure 2.

**Figure 3:**
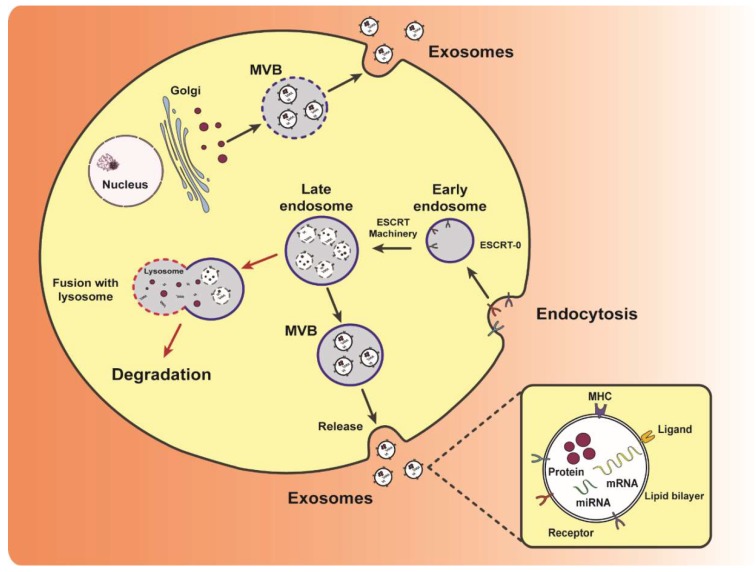
Schematic representation of exosome biogenesis and release. Biogenesis of exosomes via the ESCRT-dependent and –independent pathways are depicted. ESCRT-dependent biogenesis begins with the inward budding of the cell membrane with aid from ESCRT-0 to form early endosomes. The other ESCRT complexes aid in the packaging of exosome contents into late endosomes. These endosomes can either fuse with lysosomes to be degraded or develop into MVBs and release exosomes into the extracellular space. ESCRT-independent biogenesis includes packaging of proteins from the Golgi into exosomes within MVBs and released into the extracellular space. A wide variety of cargo can be encapsulated within exosomes, such as mRNA, miRNA, proteins.

Exosome biogenesis begins with inward budding of the cellular plasma membrane to form early endosomes. Inward budding of this endosomal membrane then forms late endosomes containing intraluminal vesicles (Figure 3). These endosomes are referred to as multivesicular bodies (MVBs) and contain multiple vesicles carrying various proteins, lipids and nucleic acids of the parent cell. Matured MVBs can either fuse with the plasma membrane (secretory MVBs) or lysosomes (degradative MVBs) [6]. The fusion of MVBs with the cell plasma membrane releases these vesicles into the extracellular milieu as exosomes [7] (Figure 3). MVBs can also fuse with lysosomes and subsequently be degraded [8]. Other unconventional pathways of biogenesis include the release of exosomes from secretory MVBs generated by bypassing the Golgi apparatus [9,10]. However, MVBs are not the only mechanism by which cells release exosomes into the extracellular space. Exosomes can also be secreted via budding instead of MVBs. For example, Jurkat T-cells have been shown to secrete outward budding vesicles enriched in exosomal protein CD63 [11].

The biogenesis of exosomes in MVBs can be delineated into two major pathways: (1) endosomal sorting complex required for transport (ESCRT)-dependent and (2) ESCRT-independent pathways [12,13,14,15]. The ESCRT-dependent pathway includes four membrane-bound complexes: ESCRT-0, ESCRT-I, ESCRT-II and ESCRT-III. These complexes have separate functions in the ESCRT pathway. In general, the pathway directs ubiquitinated proteins onto the surface of the endosome and is responsible for the budding of exosomes into the interior of the endosome, forming MVBs [16]. Other proteins have also been identified to be crucial to ESCRT-dependent exosome biogenesis. A study by Baietti et al. highlighted the role of the membrane protein syndecan and cytosolic protein syntenin in exosome production. The interaction between these proteins and ESCRT protein Alix was proven to be essential for the production of exosomes [17]. ESCRT-independent pathways in the biogenesis of exosomes involving lipids, tetraspanins and other proteins [18,19] have also been demonstrated. Indeed, Trajkovic et al. observed that ESCRT proteins were not involved in the biogenesis of proteolipid-containing exosomes [20]. Rather, they found an enrichment of ceramide (a sphingolipid) on the surface of MVBs which triggers inward budding to produce exosomes. Both ESCRT-dependent and –independent pathways have been found to require participation of cytoskeletal components (actin, microtubules), motor proteins (kinesins, myosins), and attachment proteins (SNAREs) [21].

The molecular profiles of exosomes produced through their biogenesis resembles that of the parental cell and contain endocytic proteins [8,22,23]. The composition of exosomes consists of proteins, lipids, genomic DNA, mitochondrial DNA, mRNA, microRNA and long non-coding RNA. Efforts have been made to compile a depository of exosomal proteins, RNAs and lipids from several studies into online databases such as ExoCarta [24] and Vesiclepedia [25]. Exosomal proteins include proteins identified from the cytosol, endosomes and the plasma membrane. They contain very small amounts of proteins that are of nuclear, mitochondrial, Golgi-apparatus or endoplasmic-reticulum origin [5]. Although the contents of exosomes depend highly on their parental cells, there are also ubiquitous cytosolic proteins that can be found across different types of exosomes. These ubiquitous proteins include cytoskeletal proteins (tubulin, actin and actin-binding proteins), exosome biogenesis proteins, signal transduction proteins (heterotrimeric G proteins and protein kinases, 14-3-3 proteins), metabolic proteins (pyruvate and lipid kinases, peroxidases, and enolase-I) and heat shock proteins (constitutive form of HSP90 and HSP70) [26]. In addition, these proteins also aid in antigen presentation on the surface of exosomes by binding to antigen peptides and loading these peptides onto MHC molecules [27]. Interaction with MHC molecules is aided by tetraspanins, a family of proteins that are found in abundance in exosomes. Members of the tetraspanin family (CD9, CD63, CD81 and CD82) are involved in many aspects of exosome biogenesis and function.

The exosomal membrane consists of proteins related to the endosomal membrane transport and fusion pathway (RAB, GTPases, flotillin and annexins), and the ESCRT pathway (TSG101, gag proteins and ALIX) [28]. In addition, the surface of exosomes can also contain immunoglobulin-family members (intercellular adhesion molecule 1; ICAM1), transmembrane proteins (α- and β-chains integrins), cell surface peptidases and milk-fat-globule EGF-factor VIII (MFGE8)/lactadherin [29]. FACS analysis of exosomes and their parental cell surface markers revealed that the protein composition of the exosomal membrane is markedly different from the plasma membrane as they lack membrane proteins of their parental cells such as CD28, CD40L and CD45 (T-cell-derived exosomes), Fc receptors (DC-derived exosomes), and transferrin receptor (B-cell-derived exosomes) [30]. The lipid content of exosomes include phosphatidylcholine, diglycerides, ceramides, sphingomyelin, ganglioside GM3, and cholesterol [31,32,33]. Exosomes from different parental cells have slightly different lipid contents. Platelet-derived exosomes were observed to contain low levels of lipid molecules such as phosphatidylserine on their surface [34], while B-cell-derived exosomes were observed to contain lyso-bis-phosphatidic acid, a lipid that is found in mature endosomes [35]. Both lipid proteins and phospholipases play an important role in exosome structure, packaging and function [36]. Exosomes have also been reported to be enriched in RNAs consisting of miRNAs, small non-coding RNAs (piRNA, snRNA, snoRNA, scaRNA, Y RNA), natural antisense RNAs, tRNAs and their fragments, mRNAs, and long non-coding RNAs [37]. The presence of RNA in exosomes was first reported by Valadi et al. who coined the term “exosomal shuttle RNA” to describe functional RNAs that could be internalized by the recipient cell [38].

**Figure 4:**
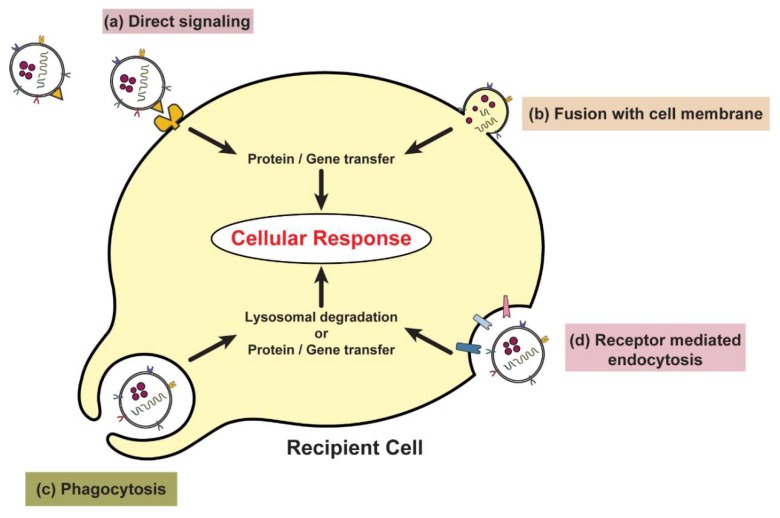
Exosome interaction and internalization by recipient cells. Interaction and/or internalization of exosomes by target cells lead to activation of various signaling pathways and transfer of exosomal contents, culminating in a cellular response. Exosomes may interact with target cells by several pathways depending on their contents and the presence of specific receptors on their surface. (**a**) Direct signaling of exosomes does not involve uptake of exosome contents into the recipient cells. Exosomes present signaling molecules on its surface that when in contact with recipient cells, illicit a response in the recipient cell (such as interaction of antigen present on exosomes with T-cells, stimulating an immune response). (**b**) Fusion with cell membrane releases exosomes contents directly into the cytoplasm. (**c**) Phagocytosis of exosomes is performed by specific cell types such as macrophages. (**d**) Receptor mediated endocytosis requires a surface ligand-receptor pair between exosomes and the recipient cell to facilitate uptake of exosomes into the cell.

Once exosomes are secreted, they travel through the extracellular millieu and get taken up by cells via (a) direct signalling through surface molecules, (b) fusion with target cell membrane, (c) receptor-dependent endocytosis and (d) the phagocytosis of opsonized exosomes (Figure 4) [39]. Exosomes attach to the plasma membrane via adhesion-associated proteins found on their surface, such as tetraspanins, SNAREs and integrins [40]. Exosome internalization can also depend on other proteins, such as actin and phosphatidylinositol 3-kinase, but the exact mechanisms for the internalization of exosomes remain unclear. Tian et al. have studied the movement of exosomes through the plasma membrane as well as within the cytoplasm of recipient PC12 cells using real-time fluorescence microscopy [41]. They found that exosomes were shuttled along the cytoskeleton or diffuse into the cytoplasm as their contents were unloaded. In a bid to find the most preferred pathway for exosome internalization, Feng et al. studied exosomes derived from K562 (chronic myelogenous leukemia) or MT4 (human T-cell line) cells and found that these were more efficiently internalized by phagocytes than by non-phagocytic cells [42]. Following cellular uptake by phagocytosis or endocytosis, exosome-delivered cargo were either incorporated into a cell, thereby effecting a biological change, or were directed to lysosomes to be degraded [39,41].

## 3. Exosomes in the Tumor Microenvironment

Direct interaction between tumor cells and the tumor microenvironment (TME) is crucial for cancer progression [43]. Many studies have unraveled the paracrine signals of various cytokines, chemokines or growth factors and their receptors as a key means of cell-cell communication within the TME and exosomes have recently emerged as another mechanism to mediate this intercellular communication. Exosomes released into the TME are a combination of exosomes secreted from cancer cells, stromal cells (including immune cells), and mesenchymal cells [44]. Such heterogeneity in exosomes generates a unique TME and contributes to the diversity of TME of different cancers. Exosomes are important players in regulating the immune response to form a pro-tumorigenic niche for cancer progression [45]. Exosomes containing active proteases are also capable of degrading extracellular matrices (ECM), thus contributing to ECM remodeling and promoting cancer cell migration and invasion [46]. Exosome secretion in cancer cells is also enhanced under hypoxic conditions [47,48], resulting in increased tumor invasiveness and stemness [49]. Here, we take a closer look at the role of exosome-derived proteins and RNAs.

### 3.1. Exosomal Proteins

Intercellular transfer of oncoproteins via exosomes has been proposed to facilitate tumor growth and metastasis. Peinado et al. demonstrated that highly metastatic melanoma cells secrete exosomes that increased the growth and metastasis of primary tumors, and programmed bone marrow progenitors towards a pro-angiogenic phenotype via receptor tyrosine kinase MET [50]. In another study, Beckler et al. surveyed the contents of exosomes secreted by mutant KRAS colon cancer cells and found that they contained oncogenic proteins including mutant KRAS which can be transferred to and promote growth of wild-type KRAS cells [51]. Exosomes secreted by prostate cancer cells were reported to transfer oncogenic proteins (Ras superfamily of GTPases), mRNAs (H-ras and K-ras) and oncomiRNAs (miR-125b, miR-130b and miR-155), and induced oncogenic reprogramming in adipose-derived stem cells (ASCs) [52]. Exosomes derived from stromal cells were also implicated in tumorigenesis. A study by Luga et al. reported that CD-81 positive exosomes derived from cancer-associated fibroblasts were able to promote breast cancer cells’ motility, metastasis and protrusive activity [53].

Cancer-cell derived exosomes have also been found to contain factors that may enhance angiogenesis. Park et al. demonstrated by proteomic analysis that exosomes derived from hypoxic tumor cells contained abundant angiogenic proteins [54]. Another study on hypoxic tumor cells showed that hypoxic glioblastoma cells secrete exosomes that were enriched in hypoxia-regulated proteins, such as matrix metalloproteinases (MMPs), platelet-derived growth factors (PDGFs), Interleukin-8 (IL-8), caveolin 1 and lysyl oxidase, and mRNAs, and were able to induce angiogenesis [55]. In addition, certain non-angiogenic factors present in tumor exosomes may also induce pro-angiogenic phenotype by modulating angiogenesis signalling pathways. A study by Nazaranko et al. showed that exosomes-mediated transfer of cell surface tetraspanin Tspan8, a non-angiogenic factor, resulted in increased endothelial cell proliferation, migration, sprouting and maturation of endothelial cell progenitors [56]. Another example of non-angiogenic protein would be oncogenic EGFR which was observed to be present on the surface of exosomes and taken up by endothelial cells and induced vascular endothelial growth factor (VEGF) expression and VEGFR2 signalling in endothelial cells [57].

Exosomes are critical mediators of the communication between cancer cells and immune cells. While exosomes can function to activate immune response and exert anti-tumor activities [58,59,60,61], they may also have immunosuppressive functions. Tumor exosomes containing TGFβ1 skew immune response away from cytotoxic mechanisms towards regulatory T cell response thereby contributing to tumor immune evasion [62]. Cancer cell-derived exosomes carrying NKG2D (NK group 2, member D) ligands downregulate NKG2D expression in NK cells and CD8+ T cells, resulting in suppression of their cytotoxic function [63]. Additionally, FasL-bearing exosomes from melanoma cells were found to induce apoptosis of T cells [64]. Collectively, these data suggest that tumor exosomes are able to enhance tumor growth by transmitting factors promoting tumorigenic behavior such as invasion and angiogenesis, while impeding the immune system and driving immune evasion of the tumor.

### 3.2. Exosomal Messenger RNA

Tumor-derived exosomes also carry various mRNAs that have pro-tumorigenic functions. Skog et al. reported that exosomes generated from glioblastoma tumor cells contained mRNAs related to tumor progression, such as proliferation and immune evasion. These mRNAs were observed to be translated into functional protein when the exosomes were taken up by recipient cells, suggesting that glioblastoma tumor cells have the potential to transform their tumor microenvironment via secretion of these exosomes [65]. In another study, Hong and colleagues profiled the transcriptome of exosomes derived from SW480 colorectal cancer cells and identified 11,327 mRNAs related to tumorigenesis. They also observed that treatment of endothelial cells with these exosomes could stimulate endothelial cell proliferation [66]. Gutkin et al. observed that exosomes were able to transfer hTERT mRNA, the transcript of the telomerase reverse transcriptase, from cancer cells into telomerase-negative fibroblasts. The transcript was translated into active enzyme in the recipient cells, allowing for these non-malignant fibroblasts to express telomerase, a key characteristic of cancer cells [67]. Exosome-derived mRNAs have also been involved in conferring drug resistance. A study by Soldevilla et al. showed that exosomes derived from colon cancer cell line HCT116 were enriched in ΔNp73 mRNA, which when transferred to recipient cells promoted their proliferation and their resistance to oxaliplatin, a chemotherapeutic drug [68].

Excitingly, mRNA levels from exosomes may also serve as surrogates in monitoring disease response in the host. Muller et al. obtained exosomes purified from the pre/post vaccination plasma of patients with gliomas in an anti-tumor vaccine Phase I/II clinical trial and compared their mRNA expression levels of 24 immunoregulatory genes [69]. They reported a significant decrease in the expression of IL-8, TGF-β, TIMP-1 and ZAP-70 in post-vaccination patients, reflecting an anti-tumorigenic state as these genes are known to be involved in angiogenesis, immune regulation and related to clinical outcome in gliomas.

### 3.3. Exosomal Microrna

MicroRNA (miRNA) profiles correlating to disease states have been detected in exosomes circulating in the host and have been found in saliva [70], blood plasma and serum [71]. The miRNA profile of these exosomes were found to mirror that of the parental tumors but was absent in healthy individuals, suggesting that the miRNA profile of circulating exosomes could constitute as possible biomarker for disease progression [72]. Examples of this can be found in colorectal cancer patients where the miRNA profile was found to differ based on the stages of the disease and its subsequent prognosis [73,74]. The same phenomenon was also observed in several other malignancies, including lung cancer [75,76], ovarian cancer [77], prostate cancer [78] and esophageal squamous cell carcinoma [79].

In vitro and mouse studies have identified specific miRNAs in exosomes that were able to affect recipient cell states. A study by Zhou et al. observed that exosome-mediated transfer of miR-105 from metastatic breast cancer cells to endothelial cells affected the integrity of vascular barriers, contributing to metastasis [80]. Another miRNA, miR-92a, a member of the miR-17-92a cluster, was observed to be secreted by leukemia cell line via exosomes and transferred to an endothelial cell line, affecting its cell formation and tube migration [81]. Exosomes were also reported to maintain oncogenic potential by transporting tumor-suppressing miRNAs out of the cell. A study on gastric metastatic cell line revealed that cells selectively release members of let-7 miRNA family, functioning mainly as tumor suppressors, into extracellular environment, maintaining a tumorigenic cell state [82]. Another study using exosomes derived from breast cancer cells and patient serum observed that cancer cells secreted exosomes contain RISC loading complex, allowing for the processing of pre-miRNA into mature miRNA within the exosomes themselves [83].

## 4. Exosomes as Components of the Senescence-Associated Secretory Phenotype

Mammalian cells can enter cellular senescence, a state of stable cell cycle arrest, when faced with cellular stress and irreparable damage [1]. Although long thought to serve as a tumor-suppressive mechanism, senescent cells can also paradoxically mediate pro-tumorigenic function via their senescence-associated secretory phenotype (SASP) production [84,85]. The SASP consists of a myriad of proteins such as inflammatory cytokines, chemokines, growth factors, matrix metalloproteinases, and affect neighbouring cells and the tumor microenvironment. Below we take a closer look at SASP in cancers and the role of exosomes as pro-tumorigenic components of the SASP.

### 4.1. The Senescence-Associated Secretory Phenotype

In addition to an intrinsic cell-autonomous tumor-suppressive mechanisms [1], SASP components such as IL-6 and IL-8 have been shown to reinforce senescence in an autocrine manner [86,87]. The SASP is also regulated by transcription factors nuclear factor-κB (NF-κB) and CCAAT/enhancer-binding protein beta (C/EBP-β) which modulate inflammatory response [86,87]. In addition, it has been shown that following DNA damage, GATA4 is stabilized and activates NF-κB, which initiates the SASP and facilitates senescence induction [88]. Surprisingly, inactivation of p53 in senescent cells causes hyperexpression of SASP, indicative of negative regulation of SASP by p53 [89]. While SASP factors can exert tumor suppression by reinforcing senescence (autocrine) or inducing senescence in neighbouring cells (paracrine) [86,90], these factors can also promote tumorigenesis, via a cell non-autonomous manner [89], in neighbouring non-senescent cells. Studies describing this cell non-autonomous pro-tumorigenic function include epithelial-mesenchymal transition and invasion, tumor vascularization, immune surveillance, and abnormal tissue morphology, mediated by proinflammatory cytokines IL-6 and IL-8, metalloprotease MMP3 and VEGF among others [87,89,91,92,93,94,95].

Various studies have shown the ability of SASP factors to establish immunosuppressive microenvironments, thereby promoting tumor growth [96,97]. For example, work from the Alimonti lab had previously shown that temporary and selective inactivation of tumor suppressor phosphatase and tensin homolog (PTEN) could lead to hyperactivation of a signalling pathway that led to senescence [98]. Subsequent work reported that activation of Janus kinase-2/signal transducer and activator of transcription-3 (Jak2/Stat3) pathway in PTEN-null senescent prostate tumors established an immunosuppressive tumor microenvironment thereby leading to its growth and chemoresistance. Combinatorial treatment with docetaxel and a JAK2 inhibitor in PTEN-null mice reprogrammed the SASP and enhanced drug response of docetaxel-induced senescent cells [96]. Additionally, senescent stromal cells have also been reported to establish permissive immunosuppressive environment [97]. Here, stromal-derived SASP factor IL-6 recruited myeloid suppressor cells and blocked anti-tumor T-cell response.

### 4.2. Exosomes as Pro-Tumorigenic SASP

Recent evidence has demonstrated that exosomes constitute part of the SASP and can mediate paracrine signaling effects on the microenvironment [99,100]. Indeed, several studies have demonstrated that secretion of exosomes is increased under conditions of cellular stress such as oxidative stress [101], telomere attrition [4], irradiation [102] and hypoxia [47], which are all conditions that induce senescence [103]. While there have been reports demonstrating exosomes as SASP in aging [100,104], their role as pro-tumourigenic SASP is only now emerging.

One of the first reports involving senescent cells revealed a significant increase in secretion of exosome-like vesicles in irradiation-induced senescent prostate cancer cells as well as in normal human diploid fibroblasts undergoing proliferative senescence [3]. The study also highlighted the role of p53 in regulating the secretion of these vesicles, suggesting a mechanistic relationship between senescence and biogenesis of these vesicles. More recently, the first-line chemotherapeutic drug paclitaxel was shown to induce senescence accompanied by enhanced secretion of exosomes in Cal51 triple negative breast cancer cells [105]. Factors of the SASP such as IL-6 receptor and full-length intercellular adhesion molecule 1 (ICAM1), were also found to be present in EVs, and based on the size and the presence of exosomal markers, these EVs are most likely exosomes [106,107]. Moreover, several studies showed that miRNAs capable of regulating cellular senescence were found to be present in exosomes [108,109]. Additionally, senescence is a crucial mechanism for maintenance of tissue homeostasis. A study by Takahashi and colleagues showed that cells maintain homeostasis by removing and secreting harmful cytoplasmic DNA via exosomes, suggesting a mechanistic role of exosomes in senescence [110]. It should be noted that senescent cells are not only able to enhance exosome secretion but can also influence exosome composition [100].

Notably, senescent cells accumulate with age and exosomes secreted from senescent cells vary from that of younger cells [111]. Indeed, Mitsuhashi et al. showed that macrophage-derived serum exosomes from old subjects contained higher levels of IL-6 and IL-12 mRNAs than those from young subjects, suggesting that the difference in exosome composition might be due to the number of senescent cells in the subjects [112]. Similarly, Takasugi et al. also showed that in addition to enhanced secretion of exosome-like small extracellular vesicles (sEVs) during senescence in both normal human fibroblasts and human retinal pigment epithelial cells, there was also an altered protein composition in sEVs generated from senescent cells compared to control cells. Notably, such senescent sEVs were demonstrated to act as important players in promoting enhanced cancer cell proliferation via the Eph receptor A2 (EphA2) [4].

**Figure 5:**
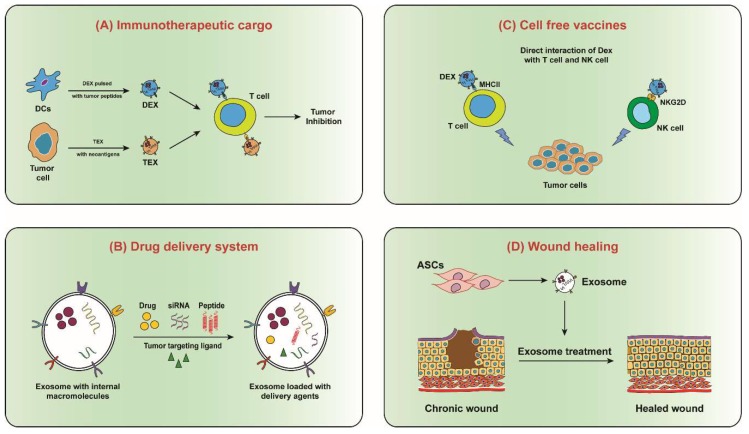
Therapeutic potential of exosomes. (**A**) Exosomes from dendritic cells (DEX) and tumour cells (TEX), carries immunogenic agents that can be transferred to tumour cell for immunomodulation. (**B**) Exosomes can be used as a drug delivery system in which versatile delivery agents can be loaded for disease prevention and treatment applications. (**C**) DEX may directly interact with and present antigen to T cells and NK cells in order to kill tumour cells. It is also possible that DEX may adhere to DC surfaces and directly present antigenic peptides in the context of MHC molecules. (**D**) Exosomes from adipose mesenchymal stem cells (ASCs) contributes to skin wound healing either via differentiation into skin-like cells, or by promoting proliferation and migration of skin cells into the injury site by secreting exosomes. ASCs secreted exosomes can be either injected onto the wounded skin area or applied on skin wound using biofilm dressings.

Additionally, our unpublished data indicate that the impact of exosomes released from mitotic-slippage induced senescent (MIS) [113] breast cancer cells can go beyond altering the biology of epithelial mammary cell by promoting cell non-autonomous inflammatory signalling in recipient non-transformed mammary epithelial cells (Jakhar et al., manuscript under review). Senescent cell-derived exosomal components were also found to increase incidence of micronuclei frequencies [114] and chromosomal instability (Takahashi and colleagues, personal communication; Jakhar et al, manuscript under review). Work is currently underway to fully characterize proteins, mRNAs and miRNAs that increase chromosomal instability and various tumorigenic phenotypes observed. Our findings lend support to the notion that exosomes from senescent cells can function as pro-tumorigenic SASP (Figure 5).

## 5. Perspectives

In this review, we have demonstrated that exosomes play pivotal roles in intercellular communication, cellular homeostasis and disease. The discovery of exosomes secreted by malignant cells afforded new hypotheses in the field especially in the study of metastasis, senescence and therapy resistance. We believe that the bioactive molecules present in the tumour microenvironment described in Section 3 may also influence cancer initiation/progression in the senescent microenvironment. Since senescent cells accumulate with age [115], we propose that exosomes derived from senescent cells could render insights into the impact of aged cells and the aged tumour microenvironment. The number of exosomes increased significantly in senescent cells compared to non-senescent cells [4]. Additionally, the number of extracellular vesicles, including exosomes, increase with age [104]. Exosomes derived from senescent cells confer pro-tumourigenic SASP and inflammation in recipient cells. Since aging is accompanied by chronic inflammation, we speculate that studying exosomes from senescent cells in the cancer context may render clues into the impact of aging on cancer initiation and progression, and therapeutic resistance. Further in-depth investigation will be needed to support the plausibility that exosomes isolated from senescent cells could contribute to cancer via biological properties that mimic the “aged” context.

**Figure 6:**
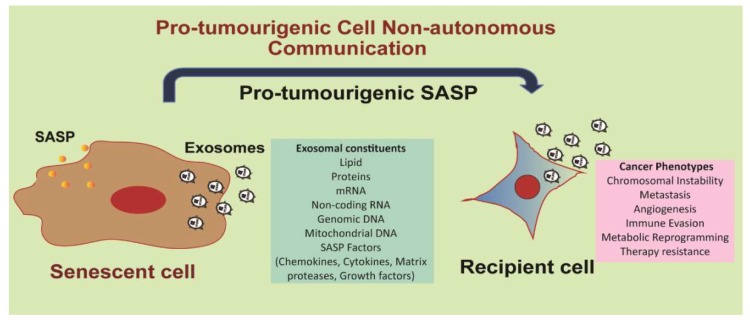
Senescent cell-derived exosomes serve as pro-tumorigenic SASP. Senescent cells usually secrete a myriad of factors such as cytokines, chemokines, matrix proteases and growth factors; this senescent secretome is termed as the senescence-associated secretory phenotype (SASP). Senescent cells produce enormous amounts of exosomes compared to normal cells. These exosomes constitute part of the SASP themselves, in addition to mediating transfer of SASP factors to recipient cells. Exosomal uptake in recipient cells can potentially result in increased chromosomal instability, metastasis, angiogenesis, immune evasion, metabolic reprogramming, and therapy resistance.

This is exciting as it provides new avenues of inhibiting cancer progression by targeting tumour-derived exosomes or inhibiting exosome biogenesis. A deeper understanding of exosome biogenesis, loading and internalization of specific cargo within senescent cells, and their subsequent uptake in recipient cells could also shed light on mechanisms behind enhanced secretion of exosomes in senescent cells. Such a study could also provide novel targets to pursue in cancer therapy. Additionally, exosomes by themselves could potentially be used as a therapeutic to deliver drugs and biomolecules (siRNA, miRNA, proteins) to alter cellular function in recipient cells and serve as attractive candidates for next-generation targeted drug delivery (Figure 6). For example, genetically-modified cells could be used to enhance secretion of exosomes with immunostimulatory functions or anti-tumorigenic miRNAs for elimination of malignant cells. Indeed, exosomes are small in size and comprise of natural components that enable them to cross biological membranes and their lipid bilayer structure protects their cargo from degradation and facilitates delivery to their target [116,117]. Figure 6 depicts various other strategies by which exosomes could be exploited for therapy. Developments in this field may offer alternative treatments especially in cancer patients that lack suitable treatment options. Additionally, exosomes in blood, saliva, urine could potentially serve as a source of specific and reliable diagnostic biomarkers for cancer.

The field, however, does face several challenges, especially in the areas of exosomal research methodologies and tools in vivo. Apart from studies on exosomes as biomarkers, the majority of exosome research in cancer has been performed in vitro. Due to the mixture of subtypes of circulating exosomes and cells that are isolated from bodily fluids, advances in exosome isolation, quantification and analysis techniques would be helpful to the study of their pathological roles in vivo. Additionally, to observe their uptake and track exosomes in animal models, in vivo studies often use exosomes extracted from in vitro cell culture. Hence, future studies focused on better elucidation of the function and influence of exosomes in the tumor microenvironment in vivo will benefit the field immensely.
